# Genetic Analysis of West Nile Virus Isolates from an Outbreak in Idaho, United States, 2006–2007

**DOI:** 10.3390/ijerph10094486

**Published:** 2013-09-23

**Authors:** Andriyan Grinev, Caren Chancey, Germán Añez, Christopher Ball, Valerie Winkelman, Phillip Williamson, Gregory A. Foster, Susan L. Stramer, Maria Rios

**Affiliations:** 1 Center for Biologics Evaluation and Research, Food and Drug Administration, Bethesda, MD 20892, USA; E-Mails: caren.chancey@fda.hhs.gov (C.C.); german.anez@fda.hhs.gov (G.A.); 2 Idaho Bureau of Laboratories, Boise, ID 83712, USA; E-Mail: ballc1@dhw.idaho.gov; 3 Creative Testing Solutions, Tempe, AZ 85282, USA; E-Mails: VWinkelman@mycts.org (V.W.); PWilliamson@mycts.org (P.W.); 4 American Red Cross, Gaithersburg, MD 20877, USA; E-Mails: Gregory.Foster@redcross.org (G.A.F.); Susan.Stramer@redcross.org (S.L.S.)

**Keywords:** *Flavivirus*, West Nile virus, genetic variation, WNV evolution, indel motifs, next generation sequencing

## Abstract

West Nile virus (WNV) appeared in the U.S. in 1999 and has since become endemic, with yearly summer epidemics causing tens of thousands of cases of serious disease over the past 14 years. Analysis of WNV strains isolated during the 2006–2007 epidemic seasons demonstrates that a new genetic variant had emerged coincidentally with an intense outbreak in Idaho during 2006. The isolates belonging to the new variant carry a 13 nt deletion, termed ID-Δ13, located at the variable region of the 3′UTR, and are genetically related. The analysis of deletions and insertions in the 3′UTR of two major lineages of WNV revealed the presence of conserved repeats and two indel motifs in the variable region of the 3′UTR. One human and two bird isolates from the Idaho 2006–2007 outbreaks were sequenced using Illumina technology and within-host variability was analyzed. Continued monitoring of new genetic variants is important for public health as WNV continues to evolve.

## 1. Introduction

West Nile virus (WNV; family *Flaviviridae*, genus *Flavivirus*) is a mosquito-borne virus that is maintained in a bird-mosquito enzootic cycle, with occasional infections of humans, horses and other animals [1]. The WNV positive RNA genome is about 11 kb in length, containing a single open reading frame (ORF) encoding one polyprotein processed into three structural and seven nonstructural viral proteins by cellular and viral proteases. It is flanked by 5′- and 3′-untranslated regions (UTR) which are involved in translation and viral RNA replication, and play an important role in genome packaging [2]. Both the 5′ and 3′UTR in the WNV genome form highly conserved secondary and tertiary structures, some elements of which are similar among mosquito-borne flaviviruses. Different functional regions have been described inside both the 5′ and 3′UTR of flaviviruses based on factors such as nucleotide content, degree of sequence conservation, occurrence of repeated sequence motifs, and predicted secondary structure [2,3,4].

**Figure 1:**
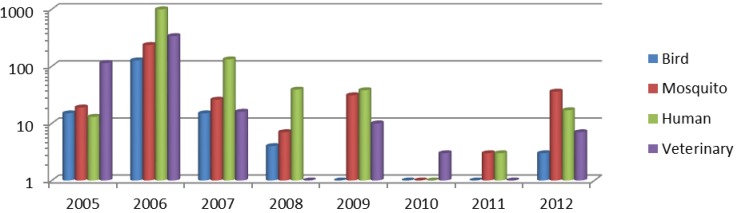
WNV infections detected in Idaho, 2005–2012. Y axis represents the number of infections in logarithmic scale and the X-axis represents years.

Since its recognition in New York City in 1999, WNV has spread throughout the United States (U.S.) and the Americas, including Canada, Mexico, the Caribbean, Central America and South America [5]. West Nile virus is now one of the most widely distributed arboviruses in the world [1,6]. Most human infections (~80%) are asymptomatic, and symptomatic infections vary from mild influenza-like illness to fatal neuroinvasive disease (~1%). The virus can be transmitted from asymptomatic viremic donors to recipients by organ transplant and by transfusion of infected blood and blood products, affecting the safety of the blood supply [7]. WNV is estimated to have infected ~4 million humans in the U.S. between 1999 and 2012, causing over 37,000 serious illnesses, including more than 16,000 neuroinvasive disease cases with 1,549 deaths reported to the Centers for Disease Control and Prevention (CDC). The first occurrence of WNV in Idaho was reported in 2003, with a single case of WNV fever and no fatalities reported. In the following years 2004 and 2005, there were respectively three and 13 human cases, including one and three cases of neuroinvasive disease, with no fatalities. In contrast, the 2006 outbreak was intense with 969 cases of disease reported to the CDC, including 115 neuroinvasive cases and 21 fatalities. The 2007 outbreak resulted in 132 cases including 10 neuroinvasive disease cases and one fatality [8]. Total numbers of human and non-human WNV infections detected in Idaho, 2005–2012, based on U.S. Geological Survey (USGS) [9] are presented in Figure 1. 

Isolates of WNV fall into up to five distinct lineages based on phylogenetic analysis, which correlate well with the geographical point of isolation [10,11,12,13]. Clade 1a of lineage I contains isolates from Africa, Europe, the Middle East, Asia, and the Americas and includes all isolates from the U.S. Phylogenetic analyses have demonstrated that WNV strains from this clade have an African origin [14]. In 2001 a new genotype, WN02, emerged in the U.S., becoming increasingly prevalent in 2002, and eventually displacing the ancestor genotype NY99 [15,16].

The WN02 genotype is characterized by 13 conserved silent nucleotide mutations and one amino acid substitution in the envelope protein, E-V_159_A, when compared with the original U.S. strain, NY99 (AF196835). This genotype became dominant in the Americas probably because of its ability to disseminate more efficiently in domestic mosquitoes as compared to the initial NY99 genotype which is believed to have come to the New World from the Middle East [17,18]. A third genotype, termed SW/WN03, is co-circulating with WN02. This genotype is characterized by two additional fixed amino acid substitutions, NS4A-A_85_T and NS5-K_314_R [19]. We performed a comprehensive phylogenetic analysis of U.S. WNV isolates from every annual epidemic between 1999 and 2011. We have found that some WNV isolates from Idaho and North Dakota collected during the 2006–2007 outbreaks form a separate cluster within genotype SW/WN03, termed MW/WN06 [20]. Analysis of MW/WN06 isolates demonstrated that they are genetically related and carry a deletion of 13 nucleotides (10,415–10,427), termed ID-Δ13, located in the variable region of the 3′UTR. 

In flaviviruses, genetic variation occurs within the linear evolutionary pathway via single base mutations and small insertions and deletions, and also infrequently by recombination [21]. WNV, like other RNA viruses, exists within each individual host as a mixture of viral particles with diverse genomes, which are also known as viral quasispecies. The genetically heterogeneous structure of viral populations allows them to promptly adapt to varying replicative environments and new hosts by selecting preexisting genetic variants with better fitness [22,23]. Minor genomes present in the viral quasispecies spectrum from the particular host in small proportion might be not recognized when a genetic study is performed using classical sequencing techniques, which normally only provides information about the major genome. However, selection acts on the entire viral swarm rather than on the individual fittest sequence. Therefore, minor genomes can potentially play an important role in adaptation to new hosts and in the virulence of circulating strains and need to be investigated.

In the past decade, a number of next generation sequencing (NGS) technologies, which are also referred to as massively parallel sequencing, high-throughput or ultra-deep sequencing, have been established. They allow determination of the primary structure of each nucleic acid molecule present in the starting material by generation of extremely large amounts of sequence information from separated individual molecules of nucleic acids [24,25]. These new methods and tools are suitable for analysis of viral quasispecies and have been used to study viruses such as SARS [26], HIV [27], hepatitis B [28], influenza [29], foot-and-mouth disease virus [30], hepatitis C and others [31]. In this article we report detailed analyses of WNV genetic variability from one human and two bird isolates from the Idaho 2006–2007 outbreaks using data from Illumina paired-end sequencing technology.

The reoccurrence of WNV outbreaks in the New World may be associated with viral adaptation through fixation of spontaneous mutations. The new genetic variant of WNV that appeared in Idaho in 2006 demonstrates the continuing evolution of the virus in North America. The emergence of new genetic variants of WNV raises issues of public health importance because emerging variants may affect the sensitivity of both screening and diagnostic assays, as well as the development of vaccines and drugs.

## 2. Experimental Section

### 2.1. Study Sample

This study included sequence analysis of the 3′UTR from 51 WNV isolates produced from human plasma specimens derived from blood donors who tested positive for viral RNA by FDA-approved nucleic acid tests (NAT) used to screen blood donations. Prior to use in our study, all human specimens were anonymized. The studied specimens were collected in the 2006–2007 epidemic seasons from the states of Colorado (CO), Idaho (ID), Illinois (IL), Kansas (KS), Maryland (MD), Minnesota (MN), Nebraska (NE), New York (NY), North Dakota (ND), South Dakota (SD), Texas (TX), and Utah (UT) under IRB-approved informed consent. In addition, one isolate from a mosquito pool and 5 avian specimens collected in Idaho in 2007 were analyzed (Table 1). The studied dataset for analysis of the variable region of the 3′UTR include the sequences of 387 WNV isolates from lineage 1 and 11 isolates from lineage 2 obtained from the GenBank database (Supplemental Table S1). 

**Table 1:** List of WNV isolates and presence of ID-Δ13 detected by RT-PCR. Y = ID-Δ13 deletion present, N = ID-Δ13 deletion absent.

#	Isolate	Host	Year	State	GenBank	ID-Δ13
			of isolation		accession no.	
1	ARC1-06	Human	2006	ID	N/A	N
2	ARC10-06	Human	2006	ID	JF957161	Y
3	ARC104-06	Human	2006	ID	N/A	Y
4	ARC105-06	Human	2006	ID	N/A	Y
5	ARC106-06	Human	2006	ID	N/A	Y
6	ARC108-06	Human	2006	ID	N/A	Y
7	ARC112-06	Human	2006	ID	N/A	Y
8	ARC13-06	Human	2006	ID	JF957162	Y
9	ARC17-06	Human	2006	ID	JF957163	Y
10	ARC19-06	Human	2006	ID	N/A	N
11	ARC22-06	Human	2006	ID	N/A	N
12	ARC23-06	Human	2006	ID	JF957164	N
13	ARC26-06	Human	2006	ID	N/A	N
14	ARC27-06	Human	2006	ID	JF957165	Y
15	ARC28-06	Human	2006	ID	N/A	N
16	ARC30-06	Human	2006	ID	N/A	N
17	ARC31-06	Human	2006	ID	N/A	N
18	ARC32-06	Human	2006	ID	N/A	N
19	ARC41-06	Human	2006	ID	N/A	N
20	ARC42-06	Human	2006	ID	N/A	Y
21	ARC57-06	Human	2006	ID	N/A	N
22	ARC60-06	Human	2006	ID	N/A	N
23	ARC61-06	Human	2006	ID	N/A	Y
24	ARC-Z-06	Human	2006	ID	N/A	N
25	ARC140-07	Human	2007	ID	JF957168	Y
26	ID7mq-07	Mosquito	2007	ID	N/A	Y
27	ID19bd-07	Avian	2007	ID	N/A	Y
28	ID20bd-07	Avian	2007	ID	N/A	Y
29	ID21bd-07	Avian	2007	ID	JF957171	Y
30	ID28bd-07	Avian	2007	ID	JF957172	Y
31	ID29bd-07	Avian	2007	ID	N/A	Y
32	ARC135-06	Human	2006	IL	N/A	N
33	ARC126-06	Human	2006	KS	N/A	N
34	ARC134-06	Human	2006	MD	N/A	N
35	ARC-W-06	Human	2006	MN	N/A	N
36	ARC-Y-06	Human	2006	MN	N/A	N
37	ARC127-06	Human	2006	NE	N/A	N
38	ARC128-06	Human	2006	NE	N/A	N
39	ARC131-06	Human	2006	NE	N/A	N
40	ARC2-06	Human	2006	NE	N/A	N
41	ARC3-06	Human	2006	NE	N/A	N
42	ARC38-06	Human	2006	NE	N/A	N
43	ARC125-06	Human	2006	NY	N/A	N
44	ARC132-06	Human	2006	SD	N/A	N
45	ARC133-06	Human	2006	TX	N/A	N
46	ARC102-06	Human	2006	UT	N/A	N
47	ARC11-06	Human	2006	UT	N/A	N
48	ARC25-06	Human	2006	UT	N/A	N
49	ARC33-06	Human	2006	UT	JF957166	N
50	BSL103-06	Human	2006	SD	N/A	N
51	BSL106-06	Human	2006	ND	JF957167	Y
52	BSL107-06	Human	2006	ND	N/A	N
53	BSL110-06	Human	2006	SD	N/A	N
54	CO2-07	Human	2007	CO	N/A	N
55	CO3-07	Human	2007	CO	N/A	N
56	CO4-07	Human	2007	CO	JF957169	N
57	CO5-07	Human	2007	CO	JF957170	N

### 2.2. Virus Isolation

Viral isolation was performed in Vero cells (ATCC # CCL-81) as described [32]. Supernatants were harvested when extensive cytopathic effect was observed, centrifuged to remove cell debris and frozen at –80 °C until further analysis. All specimens were subjected to a single passage in Vero cells.

### 2.3. RNA Extraction and Polymerase Chain Reaction (PCR)

RNA samples from viral passages were isolated from 140 µL of culture supernatants using the QIAamp Viral RNA Mini extraction kit (Qiagen, Valencia, CA, USA) according to the manufacturer’s protocol. Total RNA extracts from plasma samples were obtained from 1 mL using Trizol reagent (Invitrogen, Carlsbad, CA, USA). RNA extracts were stored at –80 °C until further analysis. Reverse transcription reactions and PCR amplification were performed for complete genome sequencing as described previously [32]. Briefly, reverse transcription reactions were performed using SuperScript III (Invitrogen) according to the manufacturer’s instructions. PCR reactions of the cDNA specimens were performed using the Hi-Fidelity PCR system (Invitrogen) according to the manufacturer’s instructions.

**Figure 2:**
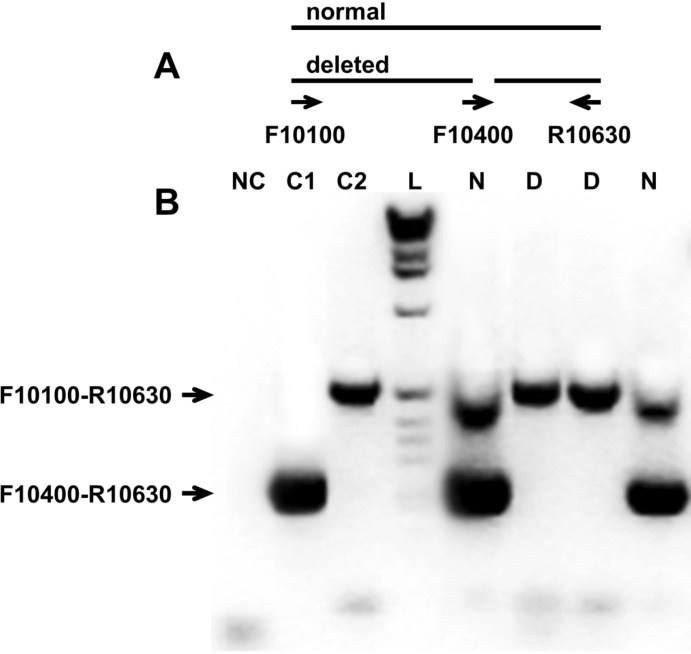
(**A**) Scheme of multiplex RT-PCR assay; (**B**) 2% agarose gel stained by ethidium bromide. NC–negative control, no viral RNA added in PCR reaction. C1 and C2–positive controls for F10400-R10630 and F10100-R10630 primers pairs respectively. L–1 kb ladder (Invitrogen). N–no deletion. D–deletion.

### 2.4. Multiplex PCR

Multiplex PCR reactions for detection of deletions in 3′UTR (Figure 2) were performed using the One-Step RT-PCR kit (Qiagen, Valencia, CA, USA) and F10100 5′-TCCATGCAGGAG GAGAGTGGATGAC; F10400 5′-CTGTAGATATTTAATCAATTGTAAATAGACAA; R10630 5′-GGGTCCTCCTTCCGAGACGGT primers to amplify fragments that allow visualization of the ID-Δ13 deletion. Amplification was performed under the following cycling conditions: cDNA synthesis at 50 °C, 30 min; denaturation at 94 °C, 15 min; PCR 30 cycles: 94 °C for 15 s, 55 °C for 30 s and 72 °C for 1 min; final extension 72 °C for 5 min. After amplification, 10 microliters of each reaction mixture were analyzed using 2% agarose gel in TAE buffer. 

### 2.5. DNA Sequencing, Assembly and Analysis

PCR products were purified after agarose gel electrophoresis using the MinElute Gel Extraction Kit (Qiagen) according to the manufacturer’s protocol, and both strands were subjected to direct sequencing using the amplifying primers and additional internal sequence primers. For validation of multiplex PCR results, sequencing reactions were performed using the F10100 and R10630 primers as described [32]. Nucleotide sequences from each isolate were aligned using the Align X program from Vector NTI (Invitrogen) and compared to the prototype NY99 (AF196835) and to previously published sequences of isolates from different regions of the U.S. and from other countries. 

### 2.6. Illumina NGS

Next generation sequencing was performed on the HiSeq 2000 platform using standard Illumina kits and protocols. We used paired-end technology to sequence both strands. Briefly, DNA samples of isolates ARC13-06, ID21bd-07 and ID28bd-07 were prepared as a mixture of the same overlapping amplicons covering the entire genomes as we used for Sanger sequencing and were then separately sheared into 200–400 bp fragments using Covaris adaptive focused acoustics process (Covaris, Woburn, MA, USA). Libraries were produced and amplified using the Paired-End DNA Sample Preparation kit according to the manufacturer’s protocols. The ends of the fragments were repaired and an A-overhang was added. Adapters specific to each sample were A-T ligated onto the ends of the fragments. Clusters were prepared using the TruSeq PE Cluster kit. All three samples were sequenced using one lane of the flow cell generating 100 bp paired-end reads. 

### 2.7. NGS Data Analysis

Preliminary NGS data analysis, conversion and trimming of read sets were performed using Illumina software CASAVA v1.8 and CLC Genomics Workbench v4. Assembly, and aligning, base calls, SNP calls, indel calls and read counts were performed using the High-performance Integrated Virtual Environment (HIVE). HIVE is a cloud-based massively parallel computational environment optimized for the storage and analysis of NGS data [33]‎. 

## 3. Results and Discussion

### 3.1. Nucleotide Mutations and Amino Acid Substitutions Identified by Sanger Sequencing

Twelve isolates from the 2006–2007 outbreaks were completely sequenced and demonstrated nucleotide divergence in the range of 0.35–0.44% compared to NY99 (AF196835). Most of the nucleotide changes were silent transitions (U↔C, A↔G) and 8 isolates shared the silent transversion A_7209_T in NS4B. Nucleotide mutations conserved in studied isolates compared to the complete genome of NY99 are shown in Table 2. 

**Table 2:** Nucleotide mutations in studied isolates compared to the complete genome of NY99 (AF196835). Non-silent mutations are shown in bold and resulted in amino acid substitutions E-V_159_A, NS4A-A_85_T and NS5-K_314_R.

Gene	prM	E								NS1						NS2A			NS2B	NS3					NS4A		
nt #	660	1320		1442		1974		2466		2661		3228		3399		3927		4146	4255	4803	6138	6238	6426		6721		6765
NY99	C	A		**T**		C		C		G		T		T		T		A	C	C	C	C	C		**G**		T
ARC10-06	T	G		**C**		T		T		A		C		C		C		G	T	T	T	T	T		**A**		C
ARC13-06	T	G		**C**		T		T		A		C		C		C		G	T	T	T	T	T		**A**		C
ARC17-06	T	G		**C**		T		T		A		C		C		C		G	T	T	T	T	T		**A**		C
ARC23-06	T	G		**C**		T		T						C				G			T	T	T		**A**		C
ARC27-06	T	G		**C**		T		T		A		C		C		C		G	T	T	T	T	T		**A**		C
ARC33-06	T	G		**C**		T		T						C				G		T	T	T	T		**A**		C
BSL106-06	T	G		**C**		T		T		A		C		C		C		G	T	T	T	T	T		**A**		C
ARC140-07	T	G		**C**		T		T		A		C		C		C		G	T	T	T	T	T		**A**		C
ID21bd-07	T	G		**C**		T		T		A		C		C		C		G	T	T	T	T	T		**A**		C
ID28bd-07	T	G		**C**		T		T		A		C		C		C		G	T	T	T	T	T		**A**		C
CO4-07	T			**C**				T										G		T	T	T	T				
CO5-07	T	G		**C**		T		T						C				G		T	T	T	T		**A**		C
**Gene**	**NS4B**												**NS5**											**3′UTR**			
**nt #**	**6936**		**6996**		**7015**		**7209**		**7245**		**7269**		**7938**		**8550**		**8621**	**8811**	**9264**	**9352**	**9660**	**10062**		**Δ13**		**10851**	
NY99	T		C		T		A		T		T		T		C		**A**	T	T	C	C	T		N		A	
ARC10-06	C		T		C		T		C		C		C		T		**G**	C	C	T	T	C		Y		G	
ARC13-06	C		T		C		T		C		C		C		T		**G**	C	C	T	T	C		Y		G	
ARC17-06	C		T		C		T		C		C		C		T		**G**	C	C	T	T	C		Y		G	
ARC23-06	C		T		C						C		C		T		**G**	C	C	T	T	C		N		G	
ARC27-06	C		T		C		T		C		C		C		T		**G**	C	C	T	T	C		Y		G	
ARC33-06	C		T		C						C		C		T		**G**	C	C	T	T	C		N		G	
BSL106-06	C		T		C		T		C		C		C		T		**G**	C	C	T	T	C		Y		G	
ARC140-07	C		T		C		T		C		C		C		T		**G**	C	C	T	T	C		Y		G	
ID21bd-07	C		T		C		T		C		C		C		T		**G**	C	C	T	T	C		Y			
ID28bd-07	C		T		C		T		C		C		C		T		**G**	C	C	T	T	C		Y		G	
CO4-07			T		C								C					C		T				N		G	
CO5-07	C		T		C						C		C		T		**G**	C	C	T	T	C		N		G	

All isolates from this study with complete viral sequences shared 12 nucleotide mutations, including one non-silent mutation in the envelope gene E-T_1442_C. The number of deduced amino acid substitutions ranged from 3 to 8, most of which were conservative substitutions. In addition to the E-V_159_A amino acid substitution in the envelope protein common to all WN02 genotype viruses, all isolates except CO4-07 shared two substitutions common to the SW/WN03 genotype, NS4A-A_85_T and NS5-K_314_R.

### 3.2. Analysis of the Variable Region of the 3′UTR

We previously reported the first identified deletion and insertion in the 3′UTR of WNV [32]. Further investigation of 3′UTR variability revealed a 13 nt deletion (10,415–10,427), named ID-Δ13, in isolates from the 2006 Idaho outbreak, in which 996 WNV symptomatic cases and 21 deaths were reported to the CDC. To investigate the penetration of the ID-Δ13 genetic variant, we designed a multiplex PCR (Figure 2) and screened 57 isolates obtained from human, bird, and mosquito specimens collected during the 2006 and 2007 epidemics. The ID-Δ13 deletion was originally identified in isolates obtained from a single passage in Vero cells. Subsequently, we tested plasma samples directly by isolating total RNA from 1 mL of plasma samples using Trizol followed by multiplex one-step PCR. Due to low plasma viral loads, only four plasma samples (ARC1-06, ARC13-06, ARC106-06 and ARC108-06) yielded sufficient RNA for downstream analysis by multiplex one-step PCR, which showed that the ID-Δ13 deletion was present in all samples except ARC1-06. The amplicons covering the region of the 3′UTR including the ID-Δ13 deletion (F10100-R10630) from these four samples were then sequenced by the Sanger method to confirm the presence of the deletion in viral RNA isolated from original plasma. There were no differences between the sequences obtained from the original plasma sample and the sequences obtained after one passage in Vero cells.

The results for the presence of ID-Δ13 deletions in the 3′UTR are shown in Table 1. All samples that had amplicon patterns corresponding to deletions were sequenced to confirm the PCR results (data not shown). Out of 31 specimens (25 humans, one mosquito, and five birds) from Idaho from 2006–2007, 18 (58%) had the ID-Δ13 deletion. The ID-Δ13 deletion was also found in one human isolate from North Dakota in 2006, but not in any isolate from other states included in this study. In addition, a deletion of 3 nt (10,499–10,501) was identified in a Colorado isolate, CO4-07. These deletions and insertion are placed within ~100 bp of the variable region of the 3′UTR located downstream of the stop codon. 

**Figure 3:**
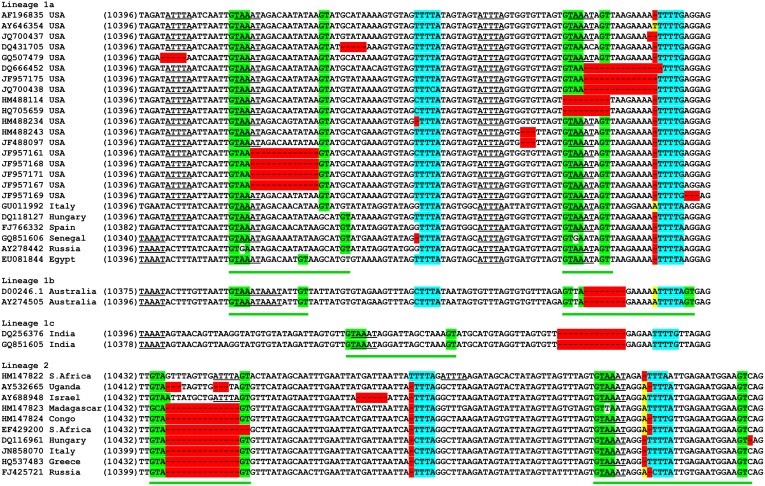
Partial nucleotide alignment of the 3′UTR of two major lineages of WNV isolated from the U.S. and other countries. GTAAATA(N)_0-29_GT repeats underlined by green lines. GTAAGT and YYYTR (Y = C/T; R = A/G) indel motifs are highlighted in green and blue colors respectively. Deletions are shown in red and insertions in yellow. AREs (ATTTA and TAAAT) are underlined. Isolates JF957161–JF957171 contained the ID-Δ13 deletion.

We used the set of 398 WNV sequences obtained from the GenBank (Supplemental Table S1) to study the variable region of the 3′UTR. The partial nucleotide alignment of the 3′UTR of two major lineages of WNV is shown in Figure 3. We observed insertions and deletions within the WNV 3′UTR occurring in the vicinity of two sequence motifs, GTAAGT and YYYTR (Y = C/T; R = A/G), which have been previously described as indel (insertion/deletion) motifs in the human genome [34]. The direct repeat (DR) GTAAATA (N)_0-29_GT appeared once at position 10,435 in lineage 1c, and twice in lineages 1a, 1b, and 2: at positions 10,413 and 10,476 in lineages 1a and 1b; and 10,434 and 10,518 in lineage 2. These DRs are flanked by the sequences GTAA and GT, which in the case of deletion of the internal part of the DR, are able to form the GTAAGT sequence. Another indel motif, YYYTR (Y = C/T; R = A/G), is located at positions 10,449 and 10,495 in lineage 1a; at positions 10,449 and 10,487 in lineage 1b; 10,481 in lineage 1c; and 10,514 and 10,558 in lineage 2. A variety of deletions and insertions in the 3′UTR of the WNV genome occurring within or near these indel motifs are illustrated in Figure 3. Analysis of this portion of the 3′UTR revealed ATTTA pentamers and complementary TAAAT pentamers located within the direct repeats described above. These pentamers are known as classic RNA instability determinants: adenosine and uridine-rich elements (ARE) found in the 3′UTR of short-lived cellular mRNAs [35].

### 3.3. Illumina NGS Data Analysis

The overlapping amplicons covering the entire genomes of ARC13-06, ID21bd-07 and ID28bd-07, which we used for common Sanger sequencing, were re-sequenced using Illumina NGS technology. NGS generated more than 80 million 100 nt reads per genome. Base and paired-end read counts estimated using the Illumina pipeline software CASAVA v1.8 are shown in Supplemental Table S2. Average quality scores per cycle ranged from 31.4–39.7. Since Phred quality scores are logarithmically linked to error probabilities, base call accuracy was in the range 99.9–99.99%. 

Base calling was performed using the sequences of each isolate identified by the common Sanger method for assembly and alignment. Approximately 1 million reads from each isolate were unaligned. Table 3 shows that there was no significant difference in the level of background sequence heterogeneity detected in the studied isolates. The average frequency of insertions and deletions ranged from 0.54–1.29% and per nucleotide variability varied by 0.82–0.89%. We found significantly more (>2 times) transitions than transversions among variable nucleotides. Substantial prevalence of transitions above transversions probably reflects the inherent bias of nucleotide misincorporation common to all known polymerases.

**Table 3:** Frequencies of mutations identified by HIVE for Illumina NGS data.

Mutation frequencies					
Isolate	Read Count, ×10^6^	Total nucleotides read, ×10^9^	Substitutions, %	Insertions, %	deletions, %
ARC13-06	80.18	8.02	0.89	0.64	1.29
ID21bd-07	91.23	9.13	0.86	0.78	1.09
ID28bd-07	83.32	8.27	0.82	0.54	1.27

The reads from each isolate were aligned by HIVE Hexagon using NY99 (AF196835) as the reference genome to perform SNP and indel calls. SNP calling results are shown in Supplemental Table S3. Illumina paired-end sequencing detected 70 mutations for isolate ARC13-06; only 46 of those, including the deletion ID-Δ13, were detectable using common Sanger sequencing. For the bird isolates ID21bd-07 and ID28bd-07, the proportions of mutations detected by NGS to mutations detected by common sequencing are 73 to 48 and 71 to 47 respectively. In the region spanning nt 10,414–10,429 where common sequencing recognized ID-Δ13, we found a broad assortment of reads which contained deletions of different sizes (1–14 nt) and some insertions (1–6 nt). Intact genomes are also present in this region in low frequencies ranging from 0.03–0.07.

All three isolates studied using NGS shared nine mutations which are not detectable by common sequencing (frequency range is shown in parentheses): E-G_2163_A (0.08–0.14); E-C_2165_G (0.08–0.14); NS4A-A_6761_G (0.06–0.09); NS4B-T_7551_A (0.10–0.16); NS5-A_9060_G (0.06–0.16); NS5-T_10142_C (0.20–0.40); NS5-T_10144_C (0.21–0.40); NS5-T_10148_A (0.20–0.41); NS5-A_10149_G (0.21–0.42). The bird isolates also shared three transversions: NS3-T_6278_G (0.085–0.088); NS3-T_6298_G (0.08–0.10); and NS4B-A_7558_T (0.08–0.14). In addition, the human isolate ARC13-06 and the bird isolate ID28bd-07 shared seven mutations in the 3′UTR: A_10432_G (0.26–0.29); C_10435_T (0.16–0.27); A_10436_T (0.17–0.27); A_10438_T (0.16–0.29); A_10440_T (0.17–0.28); G_10447_T (0.05–0.09) and T_10449_A (0.078–0.15).

The two transitions in the ORF, E-G_2163_A and NS5-A_9060_G, detected using NGS are silent. Others resulted in amino acid substitutions: E-C_2165_G in E-S_400_C; NS3-T_6278_G in NS3-V_556_G; NS3-T_6298_G in NS3-W_563_G; NS4A-A_6761_G in NS4A-E_98_G; NS4B-T_7551_A in NS4B-N_212_K; NS4B-A_7558_T in NS4B-S_215_C. A cluster of neighboring mutations in NS5, NS5-T_10142_C, NS5-T_10144_C, NS5-T_10148_A, and NS5-A_10149_G, resulted in three amino acid substitutions: NS5-V_821_A; NS5-W_822_R and NS5-I_823_K/M.

**Figure 4:**
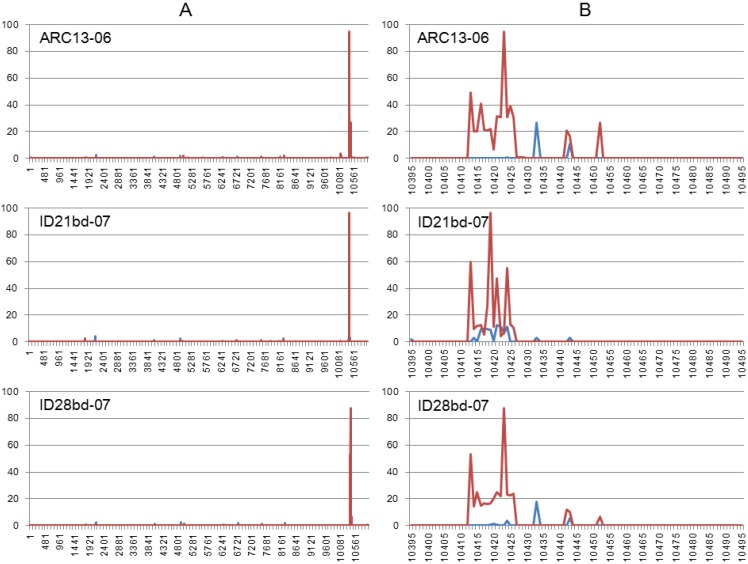
Indel profiles generated by HIVE. Y—percent of mutations; X—nucleotide number. (**A**) Indel profile of the complete genome; (**B**) Indel profile of the variable region of the 3′UTR. Deletions are shown in red, insertions in blue.

The results of the indel calls are shown in Figure 4. We found that all studied isolates demonstrated very similar indel patterns (Figure 4(A)). Detailed analysis of indel profiles of the 3′UTR variable regions (Figure 4(B)) revealed a correlation between the location of insertions and deletions and the identified indel motifs GTAAGT and YYYTR (Y = C/T; R = A/G), shown in Figure 3. 

## 4. Discussion

Several studies have examined the evolutionary dynamics and spread of WNV after its introduction into North America [15,16,19,20,32,36,37,38,39,40,41,42,43,44]. This study focuses on the analysis of a WNV genetic variant which emerged in Idaho during 2006. Our recent phylogenetic analysis of WNV isolates from the U.S. [20] demonstrates that with few exceptions, WNV strains circulating in the U.S. form phylogenetic trees that are poorly differentiated spatially and temporally. One of these exceptions is the cluster MW/WN06 within the SW/WN03 genotype, which is supported by high bootstrapping and Bayesian posterior probability values. This cluster was formed by five human WNV strains isolated from Idaho during the 2006–2007 outbreaks (ARC10-06, ARC13-06, ARC17-06, ARC27-06, ARC140-07), one human strain from North Dakota, 2006 (BSL106-06), and two avian WNV strains from Idaho collected in 2007 (ID21bd-07 and ID28bd-07) [20]. 

From 1999 through 2012, 1,243 human cases were reported to CDC from Idaho including 996 with 21 fatalities reported in 2006. Interestingly, only 13 cases of WNV infection had been reported in Idaho in 2005 before this outbreak. After the 2006 outbreak, decreasing numbers of human cases from Idaho were reported: 132 and one death in 2007; 39 and one death in 2008; 38 and two deaths in 2009; one in 2010; three in 2011; and 17 in 2012 [8]. Genetic analysis of WNV isolates from Idaho during 2006–2007 demonstrated that a new WNV variant had emerged and spread over southwest Idaho counties accounting for 58% of the 31 isolates included in this study (Table 1). These isolates carry the ID-Δ13 deletion (10,415–10,427) located at the variable region of the 3′UTR, and are genetically related. The same deletion was also identified in one human isolate from North Dakota in 2006, but not in any other isolate from that or any other state included in this study. All studied isolates from Idaho from 2007 had the ID-Δ13 deletion. The localized appearance of strains with ID-Δ13 may be due to the initial selection or introduction of one or few genetically similar viruses in the area with simultaneous and rapid spread to mosquitoes and local birds, amplifying the primarily carried genome. Human infections in that area would therefore result from one or very few local WNV colonizing genotypes, and reflect the introduction of one or very few infected vectors followed by rapid localized amplification.

Most studies on WNV evolution to date have focused on the structural genes or on the complete open reading frame. Here we have analyzed the genetic variability of WNV 3′UTR sequences using a dataset from 398 isolates from lineages 1 and 2 collected from different locations worldwide (Supplemental Table S1). Analysis of sequence alignment of WNV genomes revealed the presence of two repeats of GTAAATA (N)_0-29_GT located in the variable region of the 3′UTR (~100 nt in length, located immediately after the ORF). These repeats are present in all WNV isolates from lineages 1 and 2 available in GenBank. It is possible that conserved direct repeats in the 3′UTR of mosquito-borne flaviviruses may function as replication enhancers selected under the constraints on transmission and dissemination imposed by the particular hosts [4]. We also observed that insertions and deletions are located in the vicinity of or within the two motifs GTAAGT and YYYTR (Y = C/T; R = A/G) (Figure 3 and Figure 4). Those motifs had previously been described as indel motifs and reported to be associated with human genetic diseases [34,45,46]. Interestingly, the indel motif GTAAGT is similar to the consensus sequence at the 5′ end of introns (A/C)AG|GT(A/G)AGT in which the cleavage occurs between the G residues [47]. This observation raises suspicion that potentially the same mechanism of RNA posttranscriptional modification is acting on both host and viral genomic RNA, but that remains to be investigated. 

A detailed analysis of the variable region of the 3′UTR revealed the presence of AUUUA and complementary UAAAU pentamers in all isolates from lineage 1 and isolates that do not carry any deletion from lineage 2 (Figure 3). The AUUUA pentamer is a typical adenosine-uridine rich element (ARE) present in short-living cellular mRNAs, which usually encode proteins required only for a short period of the cell cycle, such as transcription factors, oncogenes, and cytokines [35]. Many cellular proteins bind to cellular mRNAs that contain AREs, causing either mRNA degradation (e.g., ARE/poly(U)-binding/degradation factor 1 (AUF1)) [48] or protecting the mRNA from degradation (e.g., Hu antigen R (HuR)) [49]. AREs have been found in the non-coding parts of several viral genomes including WNV [50,51]. However, the clear function of these elements in the WNV genome is yet to be determined. Studies using a reporter replicon of WNV showed that deletion of most of the 3′UTR containing the variable region did not affect translation efficiency, although the region was essential for virus replication [52]. Small deletions in the variable regions of DENV-1 and JEV do not produce any difference in replication efficiency or plaque size in mammalian or mosquito cells [53,54]. Nevertheless, some reports demonstrated that deletion of most of the variable region of the 3′UTR can affect virus replication [55,56,57,58].

The 3′UTR variable region where we identified indel motifs is located near the 5′ end of subgenomic flavivirus RNA (sfRNA). sfRNA is a small, nuclease-resistant fragment produced in significant amounts by all members of the *Flavivirus* genus and is believed to play an important role in viral replication. The production of sfRNA is a result of incomplete genomic RNA degradation after stalling of the cellular exoribonuclease XRN1 on rigid secondary RNA structures in the 3′UTR of the viral genome [59,60]. We performed preliminary studies of sfRNA using WNV genetic variants carrying the insertion and deletions that we identified, and found no significant difference when compared to isolates without deletions (data not shown), which suggests that insertions and deletions in this variable region do not play a significant role in sfRNA formation. However, the relevance and role of the identified motifs and associated genome lesions in viral replication and their evolutionary consequences remain to be explored. The relatively high rate of occurrence of mutations, deletions and insertions in the variable region of the 3′UTR suggests that positive Darwinian selection may have acted on this part of the WNV genome [61].

The appearance of WNV in the New World provides a unique opportunity to understand how an arbovirus adapts and evolves in a new replicative environment. A number of factors might be involved in evolution of WNV including the level of genetic heterogeneity of the viral populations. Selection of preexisting genetic variants in a viral quasispecies swarm can potentially play a key role in adaptation of imported viruses to domestic vectors and hosts and in dissemination of newly emerging viruses [62,63,64]. Evidence for viral population heterogeneity, where individual sequences differ from the consensus sequence, has been obtained using cloning approaches for different viruses [65,66,67,68] including WNV [69]. WNV quasispecies studies based on cloning and sequencing of the 3′ 1,159 nt of the WNV envelope coding region and the 5′ 779 nt of the WNV NS1 coding region suggests that interhost quasispecies dynamics may potentially be less significant for WNV evolution than intrahost quasispecies dynamics [69,70,71,72]. However, the cloning technique is laborious and provides only a limited resolution of the quasispecies spectrum within a sample [73]. The well-established Sanger method identifies the consensus or major viral genome present in a particular isolate, when PCR products are directly sequenced, but this method is almost uninformative about minor genetic variants represented within the quasispecies swarm. Next-generation sequencing methods produce a massive amount of sequence data which can be applied for sequencing and re-sequencing of viral genomes to obtain thorough and detailed coverage of minor variants revealing nucleotide substitutions present in only a small part of the viral population [74]. 

We have used the Illumina NGS technology for analysis of within-host genetic variability of one human and two bird isolates from the Idaho 2006–2007 outbreaks. Our data demonstrate that genetic analysis based on common Sanger sequencing of PCR products provides limited information, and it missed many non-silent mutations in the E, NS4A, NS4B and NS5 genes of studied isolates, even when those mutations were present in relatively high frequencies, such as NS5-A_10149_G (0.21–0.42). Indel call data generated by HIVE correlate with alignments based on common sequencing, and indel profiles from studied isolates demonstrate the peaks of deletion and insertion frequencies near or within identified indel motifs. However, in the location where the Sanger chromatograms showed only the 13 nucleotide deletion ID-Δ13, we also found numerous reads containing different deletions and insertions, as well as reads with intact sequences. In this study we used the same amplicons for Illumina and Sanger sequencing technologies. Those amplicons were prepared from supernatants from a single passage of original samples in Vero cells. Cultivation, as well as the downstream process used for the generation of the amplicons including synthesis of viral cDNA with subsequent PCR amplification using specific primers, has the potential for selection bias, in which some fractions of within-host viral populations may be lost. Therefore, further work is required to address this point. We are planning to compare the viral swarms present in viral isolates to those obtained from the original samples (*i.e.*, plasma, serum, mosquito pools) when sufficient starting material can be obtained. Nevertheless, our findings strongly suggest that data generated by NGS can provide new insights into WNV evolutionary dynamics at a better resolution than that previously achieved by using traditional sequencing methods.

## 5. Conclusions

The introduction of WNV into North America represents a unique opportunity to understand how an arbovirus evolves in a new replicative environment. Adaptation to domestic mosquitoes and birds through selection of preexisting genetic variants from a quasispecies swarm may have played a major role in the spread of WNV in the Americas. Genetic analysis of WNV isolates collected during the 2006–2007 epidemics reveals a new genetic variant of the virus. This variant had emerged coincidentally with an intense outbreak in Idaho during 2006. One human and two bird isolates from Idaho, 2006–2007, were re-sequenced using Illumina NGS technology and within-host genetic variability was analyzed. The NGS method produced additional data about mutations presented in minor genetic variants, which were not detectable by common direct Sanger sequencing. NGS technologies have been significantly improved in the past few years allowing for better understanding of viral evolution, fitness, emergence and transmission. Adequate surveillance based on new technologies is essential to public health since emerging mutants of pathogens could potentially affect performance of diagnostic assays, and negatively impact the development of vaccines and specific therapies. 

## Supplementary Files

Supplementary File 1 Supplementary Information (PDF, 152 KB)
