# Supplementary material for: Genetic Analysis of West Nile Virus Isolates from an Outbreak in Idaho, United States, 2006–2007
Source: Int J Environ Res Public Health. 2013 Sep 23;10(9):4486–506. doi: 10.3390/ijerph10094486 (PMC3799518; doi:10.3390/ijerph10094486)
Supplement: Supplementary File 1 — Supplementary Information (PDF, 152 KB) [file ijerph-10-04486-s001.pdf]

Genetic Analysis of West Nile Virus Isolates from an Outbreak in Idaho, United States, 2006–2007

Table S1. List of WNV strains used for the 3′UTR study.

#	GenBank accession no.	Lineage	Strain name	Host	Year of isolation	Country/State
1	EU081844	1a	Egypt 101	Human	1951	Egypt
2	AM404308	1a	PTRoxo	Mosquito	1971	Portugal
3	GQ851606	1a	ArD27875	Avian	1979	Senegal
4	AY701412	1a	96-111	Horse	1996	Morocco
5	AF260969	1a	RO97-50	Mosquito	1996	Romania
6	AY268133	1a	PaH001	Human	1997	Tunisia
7	AF481864	1a	IS-98 STD	Avian	1998	Israel
8	AF404757	1a	Italy 1998-Equine	Equine	1998	Italy
9	AF196835	1a	NY99-flamingo382-99	Avian	1999	USA/NY
10	AF202541	1a	HNY1999	Human	1999	USA/NY
11	AF206518	1a	2741	Mosquito	1999	USA/CT
12	AF260967	1a	NY99-eqhs	Horse	1999	USA/NY
13	AY842931	1a	385-99	Avian	1999	USA/NY
14	HM488125	1a	BID-V4186	Avian	1999	USA/CT
15	HM488126	1a	BID-V4187	Avian	1999	USA/CT
16	HM488127	1a	BID-V4188	Avian	1999	USA/CT
17	HM488128	1a	BID-V4189	Avian	1999	USA/CT
18	HQ596519	1a	4132	Avian	1999	USA/NY
19	HQ671706	1a	BID-V4898	Mosquito	1999	USA/CT
20	AY278441	1a	Ast99-901	Human	1999	Russia
21	AY277252	1a	LEIV-VIg99-27889	Human	1999	Russia
22	AF404753	1a	MD 2000-crow265	Avian	2000	USA/MD
23	AF404754	1a	NJ 2000 MQ5488	Mosquito	2000	USA/NJ
24	AF404755	1a	NY 2000-grouse3282	Avian	2000	USA/NY
25	AF404756	1a	NY 2000-crow3356	Avian	2000	USA/NY
26	HM488129	1a	BID-V4191	Mosquito	2000	USA/CT
27	HM488130	1a	BID-V4192	Mosquito	2000	USA/CT
28	HM488131	1a	BID-V4193	Mosquito	2000	USA/CT
29	HM488132	1a	BID-V4194	Mosquito	2000	USA/CT
30	HQ671708	1a	BID-V4900	Mosquito	2000	USA/CT
31	HQ671709	1a	BID-V4901	Mosquito	2000	USA/CT
32	HQ671710	1a	BID-V4902	Mosquito	2000	USA/CT
33	HQ671711	1a	BID-V4903	Mosquito	2000	USA/CT
34	HQ671712	1a	BID-V4904	Mosquito	2000	USA/CT
35	AY278442	1a	LEIV-Vlg00-27924	Human	2000	Russia
36	AY268132	1a	PaAn001	Horse	2000	France
37	AF533540	1a	NY_2001	Human	2001	USA/NY
38	DQ080072	1a	FL232	Avian	2001	USA/FL
39	DQ164194	1a	NY 2001 Suffolk	Avian	2001	USA/NY

40	FJ527738	1a	LSU-AR01	Avian	2001	USA/LA
41	GQ379156	1a	FL2001_67030	Avian	2001	USA/FL
42	JF920307	1a	BID-V4907	Mosquito	2001	USA/CT
43	HM488133	1a	BID-V4195	Mosquito	2001	USA/CT
44	HM488134	1a	BID-V4198	Mosquito	2001	USA/CT
45	HM488136	1a	BID-V4200	Mosquito	2001	USA/CT
46	HM488246	1a	BID-V4689	Avian	2001	USA/NY
47	HM488247	1a	BID-V4691	Avian	2001	USA/NY
48	HM488248	1a	BID-V4694	Avian	2001	USA/NY
49	HM488249	1a	BID-V4696	Avian	2001	USA/NY
50	HM756661	1a	BID-V4692	Avian	2001	USA/NY
51	HM756662	1a	BID-V4693	Avian	2001	USA/NY
52	HM756663	1a	BID-V4697	Avian	2001	USA/NY
53	HQ671697	1a	BID-V4197	Mosquito	2001	USA/CT
54	HQ671713	1a	BID-V4905	Avian	2001	USA/CT
55	HQ671714	1a	BID-V4906	Mosquito	2001	USA/CT
56	HQ671715	1a	BID-V4908	Mosquito	2001	USA/CT
57	HQ671717	1a	BID-V4910	Mosquito	2001	USA/CT
58	HQ671718	1a	BID-V4911	Mosquito	2001	USA/CT
59	HQ671719	1a	BID-V4912	Mosquito	2001	USA/CT
60	DQ411029	1a	Ast01-66	Avian	2001	Russia
61	AY289214	1a	TVP 8533	Human	2002	USA/TX
62	AY646354	1a	NY 2002	Human	2002	USA/NY
63	AY795965	1a	ARC10	Human	2002	USA/MI
64	DQ080062	1a	TWN165	Mosquito	2002	USA/LA
65	DQ164187	1a	NY_2002_Broome	Avian	2002	USA/NY
66	DQ164193	1a	NY_2002_Clinton	Avian	2002	USA/NY
67	DQ164195	1a	NY_2002_Nassau	Mosquito	2002	USA/NY
68	DQ164196	1a	GA_2002_1	Human	2002	USA/GA
69	DQ164197	1a	GA_2002_2	Human	2002	USA/GA
70	DQ164198	1a	TX_2002_1	Human	2002	USA/TX
71	DQ164205	1a	TX 2002_2	Human	2002	USA/TX
72	DQ176637	1a	TX_2002-HC	Avian	2002	USA/TX
73	GU827998	1a	Bird114	Avian	2002	USA/TX
74	JF730043	1a	BID-V5170	Mosquito	2002	USA/CT
75	HM488114	1a	BID-V4102	Mosquito	2002	USA/CT
76	HM488137	1a	BID-V4202	Mosquito	2002	USA/CT
77	HM488177	1a	BID-V4336	Avian	2002	USA/IL
78	HM488178	1a	BID-V4337	Avian	2002	USA/IL
79	HM488179	1a	BID-V4338	Avian	2002	USA/IL
80	HM488180	1a	BID-V4339	Avian	2002	USA/IL
81	HM488181	1a	BID-V4340	Avian	2002	USA/IL
82	HM488182	1a	BID-V4341	Avian	2002	USA/IL
83	HM488183	1a	BID-V4345	Avian	2002	USA/IL
84	HM488184	1a	BID-V4346	Avian	2002	USA/IL
85	HM488208	1a	BID-V4204	Mosquito	2002	USA/CT
86	HM756648	1a	BID-V4205	Avian	2002	USA/CT
87	HM756664	1a	BID-V4701	Avian	2002	USA/NY
88	HM756665	1a	BID-V4709	Avian	2002	USA/NY

89	HQ671698	1a	BID-V4203	Mosquito	2002	USA/CT
90	HQ671699	1a	BID-V4206	Mosquito	2002	USA/CT
91	HQ671720	1a	BID-V4913	Avian	2002	USA/CT
92	HQ671722	1a	BID-V4704	Avian	2002	USA/NY
93	HQ671742	1a	BID-V4343	Avian	2002	USA/IL
94	HQ705669	1a	BID-V4342	Avian	2002	USA/IL
95	DQ374650	1a	Ast02-3-717	Avian	2002	Russia
96	DQ374653	1a	Ast02-2-25	Avian	2002	Russia
97	AY660002	1a	TM171-03	Avian	2003	Mexico
98	AY712945	1a	Bird1153	Avian	2003	USA/TX
99	AY712946	1a	Bird1171	Avian	2003	USA/TX
100	AY712947	1a	Bird1461	Avian	2003	USA/TX
101	AY712948	1a	Mosquito v4369	Mosquito	2003	USA/TX
102	DQ005530	1a	BSL5-2003	Human	2003	USA/UT
103	DQ080051	1a	A-AZ-03-1623	Mosquito	2003	USA/AZ
104	DQ080052	1a	B-AZ-03-1681	Mosquito	2003	USA/AZ
105	DQ080053	1a	C-AZ-03_03-1799	Mosquito	2003	USA/AZ
106	DQ080054	1a	E-CA-03_GRLA-1260	Mosquito	2003	USA/CA
107	DQ080055	1a	F-CA-03_IMPR_102	Mosquito	2003	USA/CA
108	DQ080056	1a	G-CA-03_IMPR-1075	Mosquito	2003	USA/CA
109	DQ080057	1a	I-CA-03_Arcadia-S0331532	Avian	2003	USA/CA
110	DQ080058	1a	J-CA-03_Arcadia-S0334814	Avian	2003	USA/CA
111	DQ080059	1a	L-CA-04_SAC-04-7168	Avian	2003	USA/CA
112	DQ080070	1a	TVP9115	Avian	2003	Mexico
113	DQ164190	1a	NY_2003_Suffolk	Avian	2003	USA/NY
114	DQ164191	1a	NY_2003_Chautauqua	Avian	2003	USA/NY
115	DQ164192	1a	NY_2003_Rockland	Avian	2003	USA/NY
116	DQ164188	1a	NY_2003_Westchester	Avian	2003	USA/NY
117	DQ164189	1a	NY_2003_Albany	Avian	2003	USA/NY
118	DQ164199	1a	TX_2003	Human	2003	USA/TX
119	DQ164204	1a	CO_2003_1	Avian	2003	USA/CO
120	DQ431696	1a	03-104WI	Human	2003	USA/WI
121	GQ507472	1a	024WG-CA03OR	Human	2003	USA/CA
122	GU827999	1a	Bird1576	Avian	2003	USA/TX
123	GU828000	1a	Bird1175	Avian	2003	USA/TX
124	GU828002	1a	v4095	Mosquito	2003	USA/TX
125	GU828003	1a	Bird1881	Avian	2003	USA/TX
126	GU828004	1a	Bird1519	Avian	2003	USA/TX
127	JF920306	1a	BID-V4597	Mosquito	2003	USA/CT
128	JF920728	1a	BID-V4568	Mosquito	2003	USA/CT
129	HM488138	1a	BID-V4207	Mosquito	2003	USA/CT
130	HM488140	1a	BID-V4210	Mosquito	2003	USA/CT
131	HM488141	1a	BID-V4212	Mosquito	2003	USA/CT
132	HM488171	1a	BID-V4560	Mosquito	2003	USA/CT
133	HM488172	1a	BID-V4561	Mosquito	2003	USA/CT
134	HM488173	1a	BID-V4562	Mosquito	2003	USA/CT
135	HM488174	1a	BID-V4563	Mosquito	2003	USA/CT
136	HM488175	1a	BID-V4569	Mosquito	2003	USA/CT

137	HM488176	1a	BID-V4575	Mosquito	2003	USA/CT
138	HM488185	1a	BID-V4347	Avian	2003	USA/IL
139	HM488186	1a	BID-V4350	Avian	2003	USA/IL
140	HM488187	1a	BID-V4351	Avian	2003	USA/IL
141	HM488209	1a	BID-V4564	Avian	2003	USA/CT
142	HM488210	1a	BID-V4565	Mosquito	2003	USA/CT
143	HM488212	1a	BID-V4567	Mosquito	2003	USA/CT
144	HM488213	1a	BID-V4571	Mosquito	2003	USA/CT
145	HM488214	1a	BID-V4572	Mosquito	2003	USA/CT
146	HM488215	1a	BID-V4573	Mosquito	2003	USA/CT
147	HM488216	1a	BID-V4574	Mosquito	2003	USA/CT
148	HM488217	1a	BID-V4581	Mosquito	2003	USA/CT
149	HM488218	1a	BID-V4583	Mosquito	2003	USA/CT
150	HM488219	1a	BID-V4585	Mosquito	2003	USA/CT
151	HM488220	1a	BID-V4586	Mosquito	2003	USA/CT
152	HM488221	1a	BID-V4593	Mosquito	2003	USA/CT
153	HM488222	1a	BID-V4599	Mosquito	2003	USA/CT
154	HM488223	1a	BID-V4603	Mosquito	2003	USA/CT
155	HM488224	1a	BID-V4604	Mosquito	2003	USA/CT
156	HM488225	1a	BID-V4605	Mosquito	2003	USA/CT
157	HM488227	1a	BID-V4608	Mosquito	2003	USA/CT
158	HM488228	1a	BID-V4609	Mosquito	2003	USA/CT
159	HM488229	1a	BID-V4610	Mosquito	2003	USA/CT
160	HM488230	1a	BID-V4612	Mosquito	2003	USA/CT
161	HM488231	1a	BID-V4613	Mosquito	2003	USA/CT
162	HM488233	1a	BID-V4616	Mosquito	2003	USA/CT
163	HM488234	1a	BID-V4617	Mosquito	2003	USA/CT
164	HM488235	1a	BID-V4619	Mosquito	2003	USA/CT
165	HM488236	1a	BID-V4700	Mosquito	2003	USA/CT
166	HM488250	1a	BID-V4717	Avian	2003	USA/NY
167	HM488251	1a	BID-V4719	Avian	2003	USA/NY
168	HM756650	1a	BID-V4582	Mosquito	2003	USA/CT
169	HM756651	1a	BID-V4584	Avian	2003	USA/CT
170	HM756652	1a	BID-V4587	Mosquito	2003	USA/CT
171	HM756653	1a	BID-V4588	Mosquito	2003	USA/CT
172	HM756654	1a	BID-V4598	Mosquito	2003	USA/CT
173	HM756656	1a	BID-V4615	Mosquito	2003	USA/CT
174	HM756657	1a	BID-V4685	Mosquito	2003	USA/CT
175	HM756658	1a	BID-V4686	Mosquito	2003	USA/CT
176	HM756659	1a	BID-V4687	Mosquito	2003	USA/CT
177	HM756666	1a	BID-V4711	Avian	2003	USA/NY
178	HM756667	1a	BID-V4712	Avian	2003	USA/NY
179	HM756668	1a	BID-V4716	Avian	2003	USA/NY
180	HM756669	1a	BID-V4718	Avian	2003	USA/NY
181	HM756670	1a	BID-V4720	Avian	2003	USA/NY
182	HM756676	1a	BID-V4349	Avian	2003	USA/IL
183	HQ671700	1a	BID-V4576	Mosquito	2003	USA/CT
184	HQ671701	1a	BID-V4590	Mosquito	2003	USA/CT
185	HQ671702	1a	BID-V4595	Mosquito	2003	USA/CT

186	HQ671703	1a	BID-V4611	Mosquito	2003	USA/CT
187	HQ671704	1a	BID-V4618	Mosquito	2003	USA/CT
188	HQ671705	1a	BID-V4620	Mosquito	2003	USA/CT
189	HQ671723	1a	BID-V4715	Avian	2003	USA/NY
190	HQ705659	1a	BID-V4209	Mosquito	2003	USA/CT
191	HQ705660	1a	BID-V4714	Avian	2003	USA/NY
192	JQ700437*	1a	NY10-03	Mosquito	2003	USA/NY
193	DQ118127	1a	goose-Hungary/03	Avian	2003	Hungary
194	AY701413	1a	4.05	Horse	2003	Morocco
195	DQ080061	1a	TWN496	Avian	2004	USA/LA
196	DQ164201	1a	AZ_2004	Human	2004	USA/AZ
197	DQ431702	1a	04-216CO	Human	2004	USA/CO
198	DQ666448	1a	BSL5-2004	Human	2004	USA/AZ
199	GQ507473	1a	080WG-CA04LA	Human	2004	USA/CA
200	GQ507474	1a	091WG-CA04SB	Human	2004	USA/CA
201	HM488142	1a	BID-V4214	Mosquito	2004	USA/CT
202	HM488143	1a	BID-V4215	Mosquito	2004	USA/CT
203	HM488144	1a	BID-V4216	Mosquito	2004	USA/CT
204	HM488145	1a	BID-V4217	Mosquito	2004	USA/CT
205	HM488147	1a	BID-V4219	Mosquito	2004	USA/CT
206	HM488148	1a	BID-V4220	Mosquito	2004	USA/CT
207	HM488188	1a	BID-V4353	Avian	2004	USA/IL
208	HM488189	1a	BID-V4367	Avian	2004	USA/IL
209	HM488190	1a	BID-V4368	Avian	2004	USA/IL
210	HM488191	1a	BID-V4369	Avian	2004	USA/IL
211	HM756671	1a	BID-V4798	Avian	2004	USA/NY
212	HM756672	1a	BID-V4799	Avian	2004	USA/NY
213	HM756673	1a	BID-V4801	Avian	2004	USA/NY
214	JF488086	1a	BID-V5176	Mosquito	2004	USA/CT
215	JF488087	1a	BID-V5177	Mosquito	2004	USA/CT
216	JF488088	1a	BID-V5178	Mosquito	2004	USA/CT
217	JF488089	1a	BID-V5179	Mosquito	2004	USA/CT
218	JF488090	1a	BID-V5180	Mosquito	2004	USA/CT
219	JF488091	1a	BID-V5181	Mosquito	2004	USA/CT
220	JF488092	1a	BID-V5182	Mosquito	2004	USA/CT
221	JF488094	1a	BID-V5150	Avian	2004	USA/NY
222	JF899528	1a	BID-V4800	Avian	2004	USA/NY
223	DQ164206	1a	TX_2004	Avian	2004	USA/TX
224	AJ965628	1a	PT5.2	Mosquito	2004	Portugal
225	DQ666449	1a	GCTX1-2005	Human	2005	USA/TX
226	DQ666450	1a	GCTX2-2005	Human	2005	USA/TX
227	DQ666451	1a	BSL13-2005	Human	2005	USA/AZ
228	DQ666452	1a	BSL2-2005	Human	2005	USA/SD
229	GQ507468	1a	007WG-TX05EP	Human	2005	USA/TX
230	GQ507475	1a	099WG-CA05SB	Human	2005	USA/CA
231	GQ507476	1a	101WG-CA05SB	Human	2005	USA/CA
232	GQ507478	1a	116WG-CA05LA	Human	2005	USA/CA
233	GQ507479	1a	124WG-AZ05PI	Human	2005	USA/AZ
234	HM488115	1a	BID-V4103	Mosquito	2005	USA/CT

235	HM488116	1a	BID-V4104	Mosquito	2005	USA/CT
236	HM488117	1a	BID-V4105	Mosquito	2005	USA/CT
237	HM488118	1a	BID-V4107	Mosquito	2005	USA/CT
238	HM488119	1a	BID-V4108	Mosquito	2005	USA/CT
239	HM488120	1a	BID-V4109	Mosquito	2005	USA/CT
240	HM488121	1a	BID-V4110	Mosquito	2005	USA/CT
241	HM488149	1a	BID-V4223	Mosquito	2005	USA/CT
242	HM488150	1a	BID-V4224	Mosquito	2005	USA/CT
243	HM488151	1a	BID-V4225	Mosquito	2005	USA/CT
244	HM488152	1a	BID-V4226	Mosquito	2005	USA/CT
245	HM488192	1a	BID-V4371	Avian	2005	USA/IL
246	HM488193	1a	BID-V4373	Avian	2005	USA/IL
247	HM488194	1a	BID-V4374	Avian	2005	USA/IL
248	HM488195	1a	BID-V4375	Avian	2005	USA/IL
249	HM488196	1a	BID-V4376	Avian	2005	USA/IL
250	HM488197	1a	BID-V4377	Avian	2005	USA/IL
251	HM488198	1a	BID-V4378	Mosquito	2005	USA/IL
252	HM488252	1a	BID-V4805	Avian	2005	USA/NY
253	HM756675	1a	BID-V4806	Avian	2005	USA/NY
254	HM756677	1a	BID-V4530	Avian	2005	USA/NM
255	HQ671724	1a	BID-V4883	Avian	2005	USA/NY
256	HQ671725	1a	BID-V4885	Avian	2005	USA/NY
257	HQ671726	1a	BID-V4887	Avian	2005	USA/NY
258	JF488093	1a	BID-V5188	Mosquito	2005	USA/CT
259	JF899529	1a	BID-V4808	Avian	2005	USA/NY
260	GQ507470	1a	011WG-TX06EP	Human	2006	USA/TX
261	GQ507481	1a	142WG-NE06DO	Human	2006	USA/NE
262	GQ507482	1a	144WG-AZ06PI	Human	2006	USA/AZ
263	HM488155	1a	BID-V4229	Mosquito	2006	USA/CT
264	HM488156	1a	BID-V4230	Mosquito	2006	USA/CT
265	HM488157	1a	BID-V4231	Mosquito	2006	USA/CT
266	HM488158	1a	BID-V4232	Mosquito	2006	USA/CT
267	HM488159	1a	BID-V4233	Mosquito	2006	USA/CT
268	HM488160	1a	BID-V4355	Mosquito	2006	USA/CT
269	HM488253	1a	BID-V4553	Mosquito	2006	USA/IL
270	HM756649	1a	BID-V4354	Mosquito	2006	USA/CT
271	HQ671727	1a	BID-V4889	Avian	2006	USA/NY
272	HQ671728	1a	BID-V4891	Avian	2006	USA/NY
273	HQ671729	1a	BID-V4892	Avian	2006	USA/NY
274	JF415916	1a	TX6276	Avian	2006	USA/TX
275	JF920729	1a	BID-V5196	Mosquito	2006	USA/CT
276	JF920730	1a	BID-V5197	Mosquito	2006	USA/CT
277	JF920731	1a	BID-V5201	Mosquito	2006	USA/CT
278	JF920732	1a	BID-V5202	Mosquito	2006	USA/CT
279	JF920733	1a	BID-V5203	Mosquito	2006	USA/CT
280	JF920734	1a	BID-V5204	Mosquito	2006	USA/CT
281	JF920735	1a	BID-V5205	Mosquito	2006	USA/CT
282	JF920736	1a	BID-V5206	Mosquito	2006	USA/CT
283	JF920737	1a	BID-V5207	Mosquito	2006	USA/CT

284	JF957161	1a	ARC10-06	Human	2006	USA/ID
285	JF957162	1a	ARC13-06	Human	2006	USA/ID
286	JF957163	1a	ARC17-06	Human	2006	USA/ID
287	JF957164	1a	ARC23-06	Human	2006	USA/ID
288	JF957165	1a	ARC27-06	Human	2006	USA/ID
289	JF957166	1a	ARC33-06	Human	2006	USA/UT
290	JF957167	1a	BSL106-06	Human	2006	USA/ND
291	GQ379158	1a	ORCO0559-07	Mosquito	2007	USA/CA
292	GQ507471	1a	013WG-TX07EP	Human	2007	USA/TX
293	GQ507483	1a	148WG-CA07LA	Human	2007	USA/CA
294	HM488161	1a	BID-V4356	Mosquito	2007	USA/CT
295	HM488162	1a	BID-V4357	Mosquito	2007	USA/CT
296	HM488163	1a	BID-V4359	Mosquito	2007	USA/CT
297	HM488164	1a	BID-V4360	Mosquito	2007	USA/CT
298	HM488165	1a	BID-V4361	Mosquito	2007	USA/CT
299	HM488199	1a	BID-V4090	Avian	2007	USA/NY
300	HM488200	1a	BID-V4092	Avian	2007	USA/NY
301	HM488201	1a	BID-V4093	Avian	2007	USA/NY
302	HM488202	1a	BID-V4094	Avian	2007	USA/NY
303	HM488254	1a	BID-V4559	Mosquito	2007	USA/IL
304	HM756678	1a	BID-V4095	Avian	2007	USA/NY
305	JF415920	1a	TX7191	Avian	2007	USA/TX
306	JF488097	1a	BID-V5148	Avian	2007	USA/NY
307	JF730042	1a	BID-V5147	Avian	2007	USA/NY
308	JF920738	1a	BID-V5208	Mosquito	2007	USA/CT
309	JF920739	1a	BID-V5209	Mosquito	2007	USA/CT
310	JF920740	1a	BID-V5210	Mosquito	2007	USA/CT
311	JF920741	1a	BID-V5212	Mosquito	2007	USA/CT
312	JF920742	1a	BID-V5213	Mosquito	2007	USA/CT
313	JF920743	1a	BID-V5214	Mosquito	2007	USA/CT
314	JF920744	1a	BID-V5215	Mosquito	2007	USA/CT
315	JF920745	1a	BID-V5216	Mosquito	2007	USA/CT
316	JF920746	1a	BID-V5217	Mosquito	2007	USA/CT
317	JF957168	1a	ARC140-07	Human	2007	USA/ID
318	JF957169	1a	CO4-07	Human	2007	USA/CO
319	JF957170	1a	CO5-07	Human	2007	USA/CO
320	JF957171	1a	ID21bd-07	Avian	2007	USA/ID
321	JF957172	1a	ID28bd-07	Avian	2007	USA/ID
322	FJ766332	1a	GE-2o/V	Avian	2007	Spain
323	GQ379157	1a	DB080718-14	Avian	2008	USA/CA
324	GQ379159	1a	JPW080813-01	Squirrel	2008	USA/CA
325	HM488166	1a	BID-V4362	Mosquito	2008	USA/CT
326	HM488167	1a	BID-V4363	Mosquito	2008	USA/CT
327	HM488168	1a	BID-V4364	Mosquito	2008	USA/CT
328	HM488170	1a	BID-V4366	Mosquito	2008	USA/CT
329	HM488203	1a	BID-V4096	Avian	2008	USA/NY
330	HM488204	1a	BID-V4098	Avian	2008	USA/NY
331	HM488205	1a	BID-V4099	Avian	2008	USA/NY
332	HM488206	1a	BID-V4100	Avian	2008	USA/NY

333	HM488207	1a	BID-V4101	Avian	2008	USA/NY
334	HM488237	1a	BID-V4622	Avian	2008	USA/NY
335	HM488238	1a	BID-V4623	Avian	2008	USA/NY
336	HM488239	1a	BID-V4624	Avian	2008	USA/NY
337	HM488240	1a	BID-V4627	Avian	2008	USA/NY
338	HM488241	1a	BID-V4628	Avian	2008	USA/NY
339	HM488242	1a	BID-V4631	Avian	2008	USA/NY
340	HM488243	1a	BID-V4632	Avian	2008	USA/NY
341	HM488244	1a	BID-V4634	Avian	2008	USA/NY
342	HM488245	1a	BID-V4635	Avian	2008	USA/NY
343	HM756660	1a	BID-V4097	Avian	2008	USA/NY
344	HQ671721	1a	BID-V4625	Avian	2008	USA/NY
345	JF415921	1a	TX7558	Avian	2008	USA/TX
346	JF920747	1a	BID-V5218	Mosquito	2008	USA/CT
347	JF920749	1a	BID-V5220	Mosquito	2008	USA/CT
348	JF920750	1a	BID-V5222	Mosquito	2008	USA/CT
349	JF920751	1a	BID-V5223	Mosquito	2008	USA/CT
350	JF920752	1a	BID-V5224	Mosquito	2008	USA/CT
351	JF920753	1a	BID-V5225	Mosquito	2008	USA/CT
352	JF920754	1a	BID-V5226	Mosquito	2008	USA/CT
353	JF920755	1a	BID-V5227	Mosquito	2008	USA/CT
354	JF920756	1a	BID-V5229	Mosquito	2008	USA/CT
355	JF920757	1a	BID-V5230	Mosquito	2008	USA/CT
356	JF972636	1a	BID-V5228	Mosquito	2008	USA/CT
357	JF957173	1a	BSL173-08	Human	2008	USA/AZ
358	JF957174	1a	BSL176-08	Human	2008	USA/NV
359	JF415923	1a	M37906	Mosquito	2009	USA/TX
360	JF415924	1a	TX7827	Avian	2009	USA/TX
361	JF488095	1a	BID-V5157	Avian	2009	USA/NY
362	JF488096	1a	BID-V5159	Avian	2009	USA/NY
363	JF920758	1a	BID-V5233	Mosquito	2009	USA/CT
364	JF920759	1a	BID-V5234	Mosquito	2009	USA/CT
365	JF920760	1a	BID-V5235	Mosquito	2009	USA/CT
366	JF957175	1a	BSL2-09	Human	2009	USA/NV
367	JF957176	1a	BSL5-09	Human	2009	USA/AZ
368	JF957177	1a	BSL6-09	Human	2009	USA/NV
369	JF957178	1a	BSL11-09	Human	2009	USA/NV
370	JF957179	1a	BSL18-09	Human	2009	USA/LA
371	JF957180	1a	BSL20-09	Human	2009	USA/NV
372	JF957181	1a	BSL22-09	Human	2009	USA/SD
373	JF957182	1a	BSL24-09	Human	2009	USA/TX
374	JF957183	1a	BSL27-09	Human	2009	USA/TX
375	JF957184	1a	CO7-09	Human	2009	USA/CO
376	GU011992	1a	Ita09	Human	2009	Italy
377	JF957185	1a	BSL2-10	Human	2010	USA/AZ
378	JF957186	1a	BSL3-10	Human	2010	USA/AZ
379	JQ700438	1a	BSL4-11	Human	2011	USA/AZ
380	JQ700439	1a	BSL6-11	Human	2011	USA/MS
381	JQ700440	1a	BSL23-11	Human	2011	USA/AZ

382	JQ700441	1a	BSL24-11	Human	2011	USA/CA
383	JQ700442	1a	BSL26-11	Human	2011	USA/NY
384	D00246.1	1b	KUNCG	Mosquito	1988	Australia
385	AY274505	1b	pAKUN	Mosquito	2003	Australia
386	GQ851605	1c	G16146	Mosquito	1957	India
387	DQ256376	1c	804994	Human	1980	India
388	AY532665	2	B956	Human	1937	Uganda
389	HM147824	2	WNV from Congo	N/A	1958	Congo
390	EF429200	2	H442	Human	1958	South Africa
391	HM147822	2	WNV from South Africa	N/A	1958	South Africa
392	DQ176636	2	Madagascar-AnMg798	Avian	1978	Madagascar
393	HM147823	2	WNV from Madagascar	N/A	1988	Madagascar
394	DQ116961	2	goshawk-Hungary/04	Avian	2004	Hungary
395	FJ425721	2	Reb_VLG_07_H	Human	2007	Russia
396	HQ537483	2	Nea Santa-Greece-2010	Mosquito	2010	Greece
397	JN858070	2	Italy/2011/AN-2	Human	2011	Italy
398	AY688948	2	Sarafend	N/A	N/A	Israel

Table S2. Base and paired-end read counts estimated using CASAVA v1.8.

Sample	Seq info	Base_A	Base_C	Base_G	Base_T	Base_N	Total Bases	Read Count
ARC13-06	ID13_1	1,011,664,268	1,038,381,215	1,073,836,432	972,811,110	4,993,735	4,101,686,760	40,610,760
	ID13_2	977,015,813	1,076,620,815	1,044,948,639	1,002,361,776	739,717	4,101,686,760	40,610,760
ID21bd-07	ID21_1	1,142,509,676	1,187,206,653	1,237,138,066	1,102,143,068	5,740,976	4,674,738,439	46,284,539
	ID21_2	1,117,402,037	1,214,434,838	1,218,567,935	1,123,496,267	837,362	4,674,738,439	46,284,539
ID28bd-07	ID28_1	1,048,284,412	1,083,740,146	1,136,542,891	1,001,179,451	5,224,146	4,274,971,046	42,326,446
	ID28_2	1,011,807,605	1,121,027,049	1,110,271,129	1,031,105,485	759,778	4,274,971,046	42,326,446

Table S3. SNP call results. Each isolate were aligned by HIVE Hexagon using NY99 (AF196835) as reference genome. Mutations recognized by Sanger sequencing are highlighted in yellow. The location of the ID-Δ13 deletion is highlighted in green.

ARC13-06

nt #	NY99	SNP	Frequency	Coverage
660	C	T	0.9942	353833
1149	T	C	0.9956	271202
1320	A	G	0.9938	276477
1442	T	C	0.9967	226630
1974	C	T	0.9958	1044241
2163	G	A	0.1268	507382
2165	C	G	0.1262	505389
2181	C	T	0.9935	411207
2241	T	C	0.9961	384485
2466	C	T	0.9971	831909
2661	G	A	0.9168	949650
3228	T	C	0.9786	573847
3247	T	G	0.1139	641873
3399	T	C	0.9965	1076232
3729	T	C	0.9962	260295

3927	T	C	0.9973	1114581
4146	A	G	0.9979	2316525
4255	C	T	0.9928	486543
4347	C	T	0.9935	1264896
4441	G	A	0.9939	487178
4803	C	T	0.7523	341465
5016	A	T	0.0863	282152
5019	C	A	0.0818	279689
5024	C	G	0.0788	290735
5655	A	C	0.1711	451123
5760	A	T	0.9976	457719
6138	C	T	0.9975	185963
6238	C	T	0.9952	220673
6426	C	T	0.6128	568063
6621	T	C	0.9963	317296
6721	G	A	0.9901	163327
6761	A	G	0.0921	971792
6765	T	C	0.5448	932299
6877	C	T	0.9956	278980
6936	T	C	0.9971	680277
6996	C	T	0.9983	324174
7015	T	C	0.9964	241991
7209	A	T	0.9975	211041
7245	T	C	0.998	226650
7269	T	C	0.9953	282310
7516	T	C	0.9966	237822
7527	C	T	0.9976	519458
7551	T	A	0.128	231524
7635	A	G	0.9951	388721
7938	T	C	0.9921	171252
8550	C	T	0.9963	266956
8621	A	G	0.9962	240040
8811	T	C	0.9968	552964
9060	A	G	0.0614	219338
9264	T	C	0.9943	388508
9352	C	T	0.9946	389625
9660	C	T	0.9966	228577
9924	G	A	0.9913	333359
10062	T	C	0.996	257685
10142	T	C	0.4064	391299
10144	T	C	0.4018	390969
10148	T	A	0.4107	381677
10149	A	G	0.4182	388438
10417	A	G	0.9925	232350
10420	G	T	0.7567	207413
10421	A	G	0.9874	159772
10427	T	A	0.6553	239735

10432	A	G	0.2857	118517
10435	C	T	0.2673	124913
10436	A	T	0.2677	124737
10438	A	T	0.2852	117431
10440	A	T	0.2787	117033
10447	G	T	0.094	121064
10449	T	A	0.1545	98598
10607	T	C	0.9963	1262784
10851	A	G	0.9977	402011

ID21bd-07

nt #	NY99	SNP	Frequency	Coverage
480	C	T	0.9961	253794
660	C	T	0.9953	319802
1320	A	G	0.9947	212608
1371	C	T	0.9966	226690
1442	T	C	0.997	262265
1480	G	T	0.9962	270526
1620	C	T	0.9969	275296
1974	C	T	0.9959	734235
2163	G	A	0.1365	439995
2165	C	G	0.1357	438702
2466	C	T	0.9957	571116
2661	G	A	0.9667	1160992
2862	C	T	0.9966	386736
3228	T	C	0.99	260065
3318	T	C	0.9975	378963
3399	T	C	0.9961	661169
3649	C	T	0.9965	265700
3927	T	C	0.9948	656552
4036	C	T	0.9905	192223
4132	C	T	0.4022	1538480
4146	A	G	0.9983	640391
4255	C	T	0.9967	393254
4803	C	T	0.8676	412536
5823	C	T	0.9972	377759
5940	C	T	0.9969	392063
6138	C	T	0.998	293039
6238	C	T	0.998	549920
6278	T	G	0.0881	618297
6298	T	G	0.1017	329586
6345	C	T	0.9921	546867
6426	C	T	0.7152	1002267
6486	T	G	0.0638	524412
6672	C	T	0.9865	206814

6681	C	T	0.5882	601393
6721	G	A	0.9882	241015
6747	C	T	0.9972	2469858
6761	A	G	0.0646	1341693
6762	A	G	0.6819	1323523
6765	T	C	0.7119	736273
6936	T	C	0.9946	524101
6996	C	T	0.9987	346363
7015	T	C	0.9969	381036
7089	T	C	0.9963	376788
7209	A	T	0.9959	283346
7245	T	C	0.9967	275712
7269	T	C	0.9917	300829
7551	T	A	0.1572	673608
7558	A	T	0.1351	648157
7938	T	C	0.9946	359453
8403	A	G	0.9882	707377
8550	C	T	0.9845	389767
8621	A	G	0.9893	367629
8745	A	G	0.9892	307725
8811	T	C	0.997	419178
8886	A	G	0.9905	692438
9060	A	G	0.163	350094
9264	T	C	0.9972	356992
9352	C	T	0.9971	386101
9465	T	C	0.9958	359687
9660	C	T	0.9985	777716
10062	T	C	0.9972	278573
10142	T	C	0.2685	469113
10144	T	C	0.2642	474003
10148	T	A	0.2565	457874
10149	A	G	0.2738	454899
10393	C	T	0.9914	200605
10416	A	G	0.456	238140
10417	A	G	0.4889	234410
10420	G	A	0.5573	217991
10420	G	T	0.3293	217991
10421	A	G	0.4123	176982
10426	A	G	0.6067	180687
10427	T	A	0.2739	259133
10851	A	G	0.1985	2130258

ID28bd-07

nt #	NY99	SNP	Frequency	Coverage
660	C	T	0.9952	301337
867	T	C	0.9968	246051

1320	A	G	0.9945	245689
1442	T	C	0.9972	279319
1974	C	T	0.9962	759012
2163	G	A	0.0789	397017
2165	C	G	0.078	395552
2181	C	T	0.9937	342731
2466	C	T	0.9964	513488
2661	G	A	0.9684	937999
3228	T	C	0.9893	197880
3399	T	C	0.997	656657
3729	T	C	0.9943	299768
3927	T	C	0.996	800068
4146	A	G	0.9983	739537
4248	T	C	0.9952	309259
4255	C	T	0.9972	354708
4803	C	T	0.8752	338905
5058	T	C	0.995	252834
6039	G	A	0.9943	369017
6138	C	T	0.9981	315371
6238	C	T	0.9985	605617
6278	T	G	0.0849	475902
6298	T	G	0.083	311465
6426	C	T	0.7954	964741
6621	T	C	0.997	527386
6721	G	A	0.9904	172689
6761	A	G	0.0647	930632
6765	T	C	0.674	569787
6843	A	G	0.9901	329011
6877	C	T	0.994	532329
6931	C	T	0.9925	537879
6936	T	C	0.9942	464177
6996	C	T	0.9987	296579
7015	T	C	0.9972	264856
7209	A	T	0.9973	182506
7245	T	C	0.9976	188717
7269	T	C	0.9928	197679
7389	C	T	0.9935	183895
7527	C	T	0.9931	624647
7551	T	A	0.1005	539104
7558	A	T	0.0811	550732
7938	T	C	0.9939	298678
8550	C	T	0.987	332521
8621	A	G	0.9941	311643
8811	T	C	0.9972	397517
9060	A	G	0.0822	291168
9264	T	C	0.9973	321604
9352	C	T	0.9974	347544

9660	C	T	0.9985	508722
9924	G	A	0.9931	722492
9955	C	T	0.9979	1237531
10062	T	C	0.9974	361882
10142	T	C	0.2063	541775
10144	T	C	0.2081	529870
10148	T	A	0.1999	521478
10149	A	G	0.2114	523459
10417	A	G	0.9805	184262
10420	G	T	0.785	157993
10421	A	G	0.9672	128208
10427	T	A	0.5615	219781
10432	A	G	0.2604	157426
10435	C	T	0.1591	215764
10436	A	T	0.1674	212348
10438	A	T	0.1642	222561
10440	A	T	0.1696	224811
10447	G	T	0.0541	223440
10449	T	A	0.0775	206339
10542	A	G	0.5134	320031
10607	T	C	0.9971	2666418
10714	T	C	0.9957	994801
10851	A	G	0.9985	2278322

© 2013 by the authors; licensee MDPI, Basel, Switzerland. This article is an open access article distributed under the terms and conditions of the Creative Commons Attribution license (http://creativecommons.org/licenses/by/3.0/).
